# Association between second- and third-trimester maternal lipid profiles and adverse perinatal outcomes among women with GDM and non-GDM: a retrospective cohort study

**DOI:** 10.1186/s12884-023-05630-5

**Published:** 2023-05-05

**Authors:** Ping Shi, Jie Tang, Xiaoyan Yin

**Affiliations:** 1 Wujin Hospital Affiliated With Jiangsu University, Changzhou, Jiangsu China; 2 The Wujin Clinical College of Xuzhou Medical University, No 2 Yongning North Road, Tianning District, Changzhou, Jiangsu China

**Keywords:** Lipid profiles, Gestational diabetes mellitus, Caesarean section, Large for gestational age, Macrosomia, Neonatal unit admission

## Abstract

**Background:**

Lipid metabolism disorder during pregnancy has been reported in women with gestational diabetes mellitus (GDM). However, controversy remains regarding the relationship between maternal changes in lipid profiles and perinatal outcomes. This study investigated the association between maternal lipid levels and adverse perinatal outcomes in women with GDM and non-GDM.

**Methods:**

In total, 1632 pregnant women with GDM and 9067 women with non-GDM who delivered between 2011–2021 were enrolled in this study. Serum samples were assayed for fasting total cholesterol (TC), triglyceride (TG), low-density lipoprotein (LDL), and high-density lipoprotein (HDL) levels during the second and third trimesters of pregnancy. Adjusted odds ratios (AOR) and 95% confidence intervals (95% CI) were calculated via multivariable logistic regression analysis to determine the association of lipid levels with perinatal outcomes.

**Results:**

The serum TC, TG, LDL, and HDL levels in the third trimester were significantly higher than those in the second trimester (*p* < 0.001). Women with GDM had significantly higher levels of TC and TG in the second and third trimesters than those with non-GDM in the same trimesters, while HDL levels decreased in women with GDM (all *p* < 0.001). After adjusting for confounding factors by multivariate logistic regression, every mmol/L elevation in TG levels of women with GDM in second and third trimesters was associated with a higher risk of caesarean section (AOR = 1.241, 95% CI: 1.103–1.396, *p* < 0.001; AOR = 1.716, 95% CI: 1.556–1.921, *p* < 0.001), large for gestational age infants (LGA) (AOR = 1.419, 95% CI: 1.173–2.453, *p* = 0.001; AOR = 2.011, 95% CI: 1.673–2.735, *p* < 0.001), macrosomia (AOR = 1.220, 95% CI: 1.133–1.643, *p* = 0.005; AOR = 1.891, 95% CI: 1.322–2.519, *p* < 0.001), and neonatal unit admission (NUD; AOR = 1.781, 95% CI: 1.267–2.143, *p* < 0.001; AOR = 2.052, 95% CI: 1.811–2.432, *p* < 0.001) cesarean delivery (AOR = 1.423, 95% CI: 1.215–1.679, *p* < 0.001; AOR = 1.834, 95% CI: 1.453–2.019, *p* < 0.001), LGA (AOR = 1.593, 95% CI: 1.235–2.518, *p* = 0.004; AOR = 2.326, 95% CI: 1.728–2.914, *p* < 0.001), macrosomia (AOR = 1.346, 95% CI: 1.209–1.735, *p* = 0.006; AOR = 2.032, 95% CI: 1.503–2.627, *p* < 0.001), and neonatal unit admission (NUD) (AOR = 1.936, 95% CI: 1.453–2.546, *p* < 0.001; AOR = 1.993, 95% CI: 1.724–2.517, *p* < 0.001), which were higher than the relative risk of these perinatal outcomes in women with non-GDM. Additionally, every mmol/L increase in second and third-trimester HDL levels of women with GDM was associated with decreased risk of LGA(AOR = 0.421, 95% CI: 0.353–0.712, *p* = 0.007; AOR = 0.525, 95% CI: 0.319–0.832, *p* = 0.017) and NUD (AOR = 0.532, 95% CI: 0.327–0.773, *p* = 0.011; AOR = 0.319, 95% CI: 0.193–0.508, *p* < 0.001), and the risk reduction was not strong than that of women with GDM.

**Conclusions:**

Among women with GDM, high maternal TG in the second and third trimesters was independently associated with an increased risk of cesarean section, LGA, macrosomia, and NUD. High maternal HDL during the second and third trimesters was significantly associated with decreased risk of LGA and NUD. These associations were stronger than those in women with non-GDM, suggesting the importance of monitoring second and third-trimester lipid profiles in improving clinical outcomes, especially in GDM pregnancies.

**Supplementary Information:**

The online version contains supplementary material available at 10.1186/s12884-023-05630-5.

## Background

Gestational diabetes mellitus (GDM), a known metabolic disorder, is defined as hyperglycemia which is first diagnosed during pregnancy [1]. The global prevalence rate of GDM is increasing due to lifestyle changes and diagnostic criteria updates [2–6]. In China, GDM incidence is approximately 14.8% [7]. Additionally, GDM is significantly related to adverse perinatal outcomes [8]. Women with GDM have an increased risk of hypertension, hyperbilirubinemia, coronary heart disease, preeclampsia, and cesarean section and have a higher risk of long-term obesity and diabetes post-pregnancy [9, 10]. Moreover, GDM is strongly associated with certain neonatal outcomes, including large for gestational age (LGA) infants, fetal macrosomia, preterm birth, and postpartum hemorrhage [11, 12]. Risk factors for GDM have been reported to be connected to obesity, family history of diabetes mellitus, advanced maternal age, and gestational weight gain (GWG) [13, 14]. Dyslipidemia during pregnancy has also been reported to increase the risk of GDM [15–17].

Maternal lipid metabolism changes during pregnancy are common, physiologically necessary to ensure fetal growth [18, 19] and include moderate increases in lipids in the first trimester and significant increases in lipids in the second and third trimesters, especially in triglyceride (TG) and cholesterol levels [20, 21]. However, dyslipidemia may lead to pregnancy complications and adverse perinatal outcomes [16]. Dyslipidemia during pregnancy is significantly correlated with pregnancy-induced hypertension, GDM, preeclampsia, preterm birth, adverse birthweight outcomes, LGA neonates, cesarean delivery, and postpartum hemorrhage [19, 22–24]. However, some controversy exists regarding the correlation between dyslipidemia and pregnancy complications and perinatal outcomes. For example, several studies have shown that GDM is connected with lower maternal low-density lipoprotein (LDL) and high-density lipoprotein (HDL) levels during the second and third trimesters. Other reports found no significant differences in LDL and HDL levels between GDM and non-GDM pregnancies [25, 26]. Moreover, Wang et al. reported that TG levels increased the risk of macrosomia in non-GDM pregnancies, whereas other studies found no such association [27, 28]. Whether neonatal birth weight is positively connected with TG levels in GDM or non-GDM cases is unclear [29–31], and studies on the relationship between dyslipidemia and adverse perinatal outcomes in women with GDM and non-GDM in various trimesters are limited.

Thus, we conducted a retrospective cohort study of women with GDM and non-GDM in China to analyze the alteration of blood lipid profiles. We also comprehensively investigated the correlation between maternal changes in lipid profiles in the second and third trimesters and adverse perinatal outcomes.

## Methods

### Study participants

**Fig. 1:**
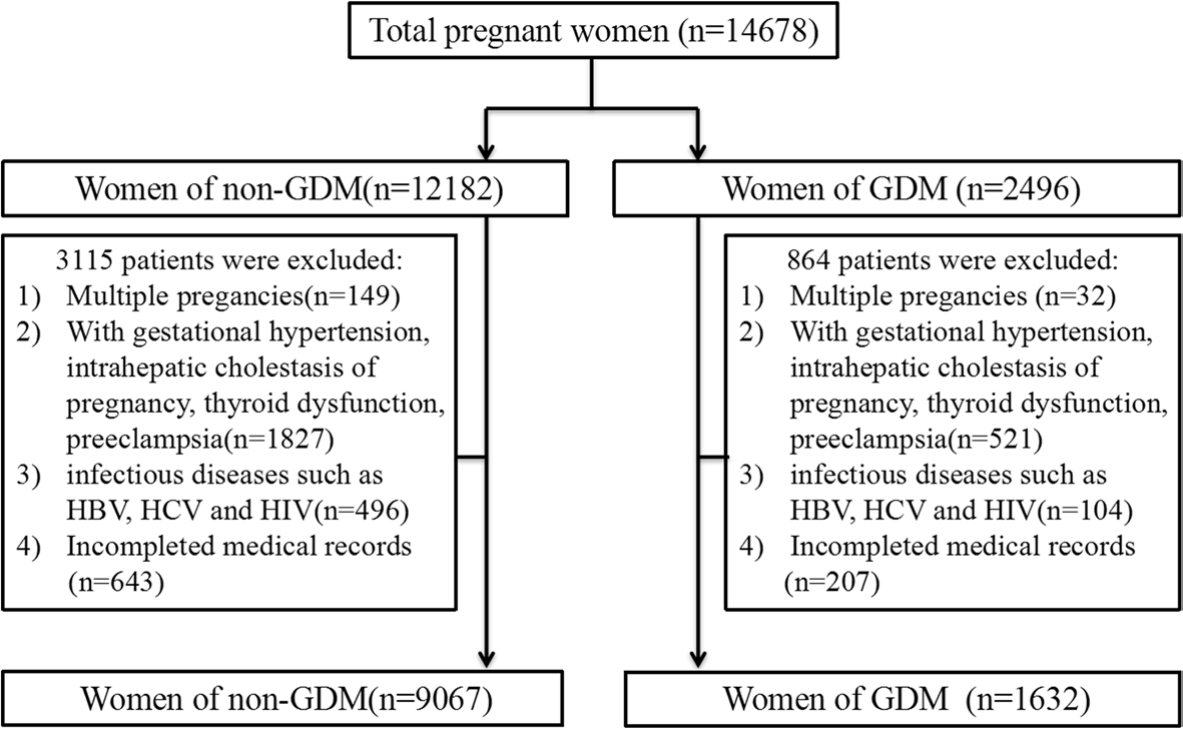
Flow diagram of study cohort

This retrospective cohort study was conducted from January 1, 2011, to December 31, 2021, at a hospital in Changzhou, Jiangsu, China. The study was approved by the ethics committee of our hospital, all procedures were performed in compliance with the Declaration of Helsinki. The inclusion criteria were as follows: pregnant women a) aged ≥ 18 years without pre-pregnancy diabetes mellitus, hypertension, heart disease, renal disease, or hepatic disease; b) singleton pregnancy and live birth; c) with complete information records. The exclusion criteria were as follows: a) multiple pregnancies; b) gestational hypertension, intrahepatic cholestasis of pregnancy, thyroid dysfunction, or preeclampsia; and c) infectious diseases such as hepatitis B virus, hepatitis C virus, and human immunodeficiency virus. In total, 14,678 pregnant women delivered at our hospital from January 1, 2011, to December 31, 2021, of which 2496 had GDM and 12,182 were non-GDM patients. However, 864 patients with GDM and 3115 patients with non-GDM were excluded after applying the exclusion criteria. Ultimately, 9067 pregnant women with non-GDM and 1632 pregnant women with GDM were included for final analysis (Fig. 1). General characteristics, including maternal age, pre-pregnancy body mass index (ppBMI), GWG, maternal education level, parity, cesarean history, in vitro fertilization, mode of delivery, and gestational age; blood fasting plasma glucose and 2-h oral glucose tolerance test (OGTT) results were extracted from the medical record system.

### Biochemical analyses

For lipid assessments, venous blood samples were collected from all pregnant women in the second (24–28 gestational weeks) and third (32–36 gestational weeks) trimesters of pregnancy following an overnight fast. Total cholesterol (TC), TG, HDL, and LDL concentrations were determined for each sample according to the manufacturer's instructions using homogeneous enzymatic colorimetric assays. All lipid measurements were performed using an automatic biochemical analyzer (Beckman AU5800, USA).

### Diagnostic criteria of GDM

All pregnant women at our hospital underwent GDM screening, with diagnostic criteria based on the criteria revised in China in August 2014. A GDM diagnosis was established if the results of a 75-g OGTT performed between 24 and 28 weeks of gestation showed any one or a combination of the following: 1) fasting blood glucose ≥ 5.1 mmol/L, 2) 1 h blood glucose ≥ 10.0 mmol/L, or 3) 2 h blood glucose ≥ 8.5 mmol/L.

### Definitions of ppBMI and GWG

The World Health Organization classification of ppBMI was used for underweight, normal weight, overweight, and obesity classifications. GWG was stratified into the following three categories according to the Institute of Medicine guidelines: appropriate, inadequate, and excessive (Supplementary Table 1).

### Adverse perinatal outcomes

Data on adverse perinatal outcomes were extracted from the medical records system. The investigated adverse perinatal outcomes included maternal outcomes, such as cesarean delivery, premature rupture of membrane, preterm birth (delivery before 37 weeks of gestation), abruptio placentae (the placenta is completely or partially detached from the uterine wall before delivery of the fetus), and postpartum hemorrhage (blood loss ≥ 500 mL for vaginal delivery and ≥ 1000 mL for cesarean delivery within 24 h after delivery of the fetus), as well as neonatal outcomes, such as LGA (birth weight exceeded the 90^th^ percentile for gestational age, SGA (small for gestational age, birth weight fell below the 10^th^ percentile for gestational age), low birth weight (LBW, birth weight < 2500 g), macrosomia (birth weight ≥ 4000 g), and neonatal unit admission (NUD). SGA and LGA were defined based on Neonatal Birth Weight for Gestational Age and Percentile in 15 Cities in China [32].

### Statistical analysis

Continuous data with normal and non-normal distributions are described as mean ± standard deviation (SD) and median with interquartile ranges (IQR). Categorical variables are presented as n (%). The Mann–Whitney U test compared maternal lipid levels between the two groups. After adjusting for potential confounding variables (age, pre-pregnancy BMI, gestational weight gain, parity, IVF, cesarean history, and abortion history) using multivariable logistic regression, adjusted odds ratios (AOR) and 95% confidence intervals (95% CI) were calculated to express the odds ratios of the lipid profiles for adverse perinatal outcomes. Bonferroni correction (multiple comparison method) was used to compare the categorical variables among the different groups. A *p*-value < 0.05 (two-sided) indicated statistical significance, and data were analyzed using the statistical package for the social sciences (SPSS) 23.0 (Armonk, NY, IBM Corp.).

## Results

### Demographic and clinical characteristics of the study population

The process of screening the study population based on the inclusion and exclusion criteria is shown in Fig. 1. Overall, 1632 women with GDM and 9067 women with non-GDM were enrolled according to the inclusion criteria, with their general clinical characteristics shown in Table 1. The mean maternal age and pre-pregnancy BMI of women with GDM were higher than those with non-GDM, and there was a higher proportion of older (age ≥ 35 years) and women with overweight and obesity (≥ 25.0 kg/m^2^) in the GDM group. Additionally, the GDM group was more likely to have a high percentage of excessive GWG, a history of cesarean section, and IVF (All *p* < 0.01).

**Table 1:** Clinical characteristics of women with GDM and non-GDM

Characteristics	Mean ± SD or N (%)		*P*
	GDM group (*n* = 1632)	non-GDM group (*n* = 9067)	
Maternal Age (years)	33.02 ± 4.36	30.80 ± 4.53	< 0.001
20–29	425(26.04)	3835(42.30)	< 0.001
30–34	650(39.83)	3409(37.60)	
≥ 35	557(34.13)	1823(20.10)	
Pre-pregnancy BMI (kg/m^2^)	21.64 ± 2.94	20.66 ± 2.64	< 0.001
Underweight (< 18.5)	228(13.97)	1804(19.90)	< 0.001
Normal weight (18.5–24.9)	1215(74.45)	6683(73.71)	
Overweight and obese (≥ 25.0)	189(11.58)	580(6.40)	
Gestational weight gain			
Appropriate	696(42.65)	3752(41.38)	< 0.001
Inadequate	612(37.50)	3991(44.02)	
Excessive	324(19.85)	1324(14.60)	
Maternal education [*n* (%)]			
≤ Junior school	165(10.11)	917(10.11)	0.896
High school	199(12.19)	1162(12.82)	
≥ University	1268(77.69)	6988(77.07)	
Caesarean history [*n* (%)]	402(24.63)	2531(27.91)	< 0.001
Parity			
Nulliparous	861(52.76)	5495(60.60)	< 0.001
Multiparous	771(47.24)	3572(39.40)	
IVF [*n* (%)]	176(10.78)	621(6.85)	< 0.001
Mode of delivery [*n* (%)]			
Vaginal	890(54.53)	5423(59.81)	< 0.001
Cesarean	742(45.47)	3644(40.19)	
Gestational age (weeks, mean ± SD)	38.35 ± 1.75	37.58 ± 2.67	< 0.001
FPG on OGTT (mmol/L)	5.59 ± 0.83	4.12 ± 0.47	< 0.001
2-h blood glucose on OGTT (mmol/L)	8.75 ± 1.69	7.21 ± 1.92	< 0.001

*GDM* Gestational Diabetes Mellitus, *IVF* In-vitro fertilization, *FPG* Fasting plasma glucose, *OGTT* Oral glucose tolerance test

*SD* Standard deviation, *pp BMI* Pre-pregnancy body mass index, *IVF* In-vitro fertilization, *FBG* Fasting Plasma Glucose, *OGTT* Oral glucose tolerance test

### Maternal lipid profiles by trimester among participants with GDM and non-GDM

Table 2 shows notable changes in the maternal lipid profiles of second and third trimester women with GDM and non-GDM. In particular, serum TC, TG, LDL, and HDL levels increased as pregnancy trimesters advanced. Serum TC, TG, LDL, and HDL levels in the third trimester were significantly higher than in the second trimester (*p* < 0.001). Additionally, women with GDM had significantly higher levels of TC and TG in the second and third-trimesters than women with non-GDM. In contrast, HDL levels decreased in women with GDM (*p* < 0.001).

**Table 2:** Comparison of maternal lipid profiles between GDM and non-GDM women

	GDM(*n* = 1632)			Non-GDM(*n* = 9067)			P^a^	P^b^
	Second	Third	*P*	Second	Third	*P*		
TC( mmol/L)	6.13(5.63–7.04)	6.51(6.02–7.66)	< 0.001	5.59(5.09–6.39)	6.03(5.24–6.75)	< 0.001	< 0.001	< 0.001
TG( mmol/L)	2.52(1.79–2.92)	3.83(2.32–4.58)	< 0.001	2.02(1.48–2.58)	3.34(2.42–4.23)	< 0.001	< 0.001	< 0.001
LDL( mmol/L)	3.22(2.47–4.19)	3.79(2.89–4.61)	< 0.001	3.13(2.44–3.96)	3.71(2.69–4.55)	< 0.001	0.819	0.589
HDL( mmol/L)	1.79(1.51–2.25)	1.62(1.41–2.01)	< 0.001	2.11(1.75–2.43)	2.45(1.85–2.57)	< 0.001	< 0.001	< 0.001

*GDM* Gestational Diabetes Mellitus, *TC* Total cholesterol, *TG* Triglyceride, *LDL* Low density lipoprotein, *HDL* High density lipoprotein. P Comparison of serum lipids between the second and third trimester, P^a^ Comparison of serum lipids in the second trimester between GDM group and non-GDM group, P^b^ Comparison of serum lipids in the third trimester between GDM group and non-GDM group

### Association between maternal lipid profiles in the second trimester and adverse perinatal outcomes among women with GDM and non-GDM

To further evaluate the effects of the maternal lipid profiles of women with GDM and non-GDM in their second trimester on perinatal outcomes, we analyzed the association of TC, TG, LDL, and HDL levels and perinatal outcomes using multivariate logistic regression (Table 3). After adjusting for confounders, we observed that every mmol/L increase in second-trimester TG concentrations of women with GDM was associated with an increased risk of cesarean delivery (AOR=1.423, 95 % CI: 1.215–1.679, *p*<0.001), LGA (AOR=1.593, 95% CI: 1.235–2.518, *p*=0.004), macrosomia (AOR=1.346, 95% CI: 1.209-1.735, *p*=0.006), and NUD (AOR=1.936, 95% CI: 1.453-2.546, *p*<0.001), which were higher than the relative risks of these perinatal outcomes in women with non-GDM. In addition, every mmol/L increase of HDL concentrations in the second trimester was associated with a reduced risk of LGA (AOR=0.421, 95% CI: 0.353–0.712, *p*=0.007) and NUD (AOR=0.532, 95% CI: 0.327–0.773, *p*=0.011) in women with GDM, but only associated with a reduced risk of LGA (AOR=0.612, 95% CI: 0.438–0.901, *p*=0.018) in women with non-GDM. In contrast, no significant associations between TC or LDL concentrations and perinatal outcomes were noted among women with GDM and non-GDM.

**Table 3:** Association of maternal lipid profile in second trimester of pregnancy with adverse pregnancy outcomes among GDM and non-GDM group

	TC, AOR (95%CI)			TG, AOR (95%CI)			LDL, AOR (95%CI)			HDL, AOR (95%CI)		
	GDM-group	non-GDM-group	*P*	GDM-group	non-GDM-group	*P*	GDM-group	non-GDM-group	*P*	GDM-group	non-GDM-group	*P*
Maternal outcome												
Caesarean delivery	0.908(0.756–1.102)	1.034(0.798–1.432)	0.879	1.423(1.215–1.679)^***^	1.107(1.071–1.236)^*^	0.002	0.938(0.668–1.132)	0.971(0.673–1.324)	0.921	0.719(0.573–1.214)	1.146(0.873–1.532)	0.569
PROM	0.913(0.703–1.109)	0.849(.0723–1.431)	0.912	1.204(0.887–1.275)	1.021(0.865–1.342)	0.694	0.836(0.689–1.205)	1.252(0.759–1.625)	0.674	1.098(0.699–1.598)	0.913(0.651–1.517)	0.774
Preterm birth	0.764(0.671–1.122)	0.961(0.832–1.335)	0.734	1.199(0.873–1.516)	0.872(0.776–1.213)	0.453	1.124(0.793–1.089)	0.921(0.746–1.432)	0.763	0.725(0.528–1.235)	1.052(0.411–1.108)	0.482
Abruptio placentae	0.903(0.612–1.156)	1.319(0.768–1.564)	0.583	0.429(0.319–0.799)^*^	0.912(0.823–1.158)	0.023	1.103(0.593–1.624)	1.123(0.799–1.581)	0.883	0.813(0.584–1.479)	0.745(0.569–1.715)	0.832
PPH	1.084(0.803–1.124)	1.113(0.921–1.263)	0.832	0.931(0.587–1.372)	1.231(0.894–1.498)	0.328	0.979(0.702–1.316)	1.322(0.941–1.582)	0.549	1.012(0.513–2.025)	1.291(0.795–1.729)	0.799
Neonatal outcome												
LGA	1.162(0.742–2.102)	1.214(0.921–1.414)	0.794	1.593(1.235–2.518)^**^	1.213(1.081–1.764)^*^	0.012	1.589(0.794–2.212)	1.453(0.799–1.756)	0.923	0.421(0.353–0.712)^**^	0.612(0.438–0.901)^*^	0.017
SGA	0.823(0.513–1.254)	0.941(0.751–1.328)	0.802	1.029(0.781–1.703)	1.216(0.879–1.763)	0.332	1.489(0.829–2.587)	1.463(0.912–2.031)	0.996	0.876(0.512–3.136)	0.796(0.405–3.489)	0.913
Macrosomia	0.906(0.624–1.246)	1.231(0.832–1.521)	0.495	1.346(1.209–1.735)^**^	1.109(0.887–1.543)	0.008	1.029(0.796–1.232)	1.293(0.773–1.841)	0.794	0.815(0.512–1.423)	0.782(0.441–1.387)	0.956
LBW	0.783(0.414–1.095)	0.816(0.712–1.234)	0.793	0.819(0.426–1.652)	0.892(0.667–1.496)	0.836	1.402(0.696–2.413)	0.992(0.759–1.569)	0.293	1.048(0.492–3.928)	0.987(0.240–4.054)	0.899
NUD	1.318(0.726–1.786)	1.236(0.814–1.697)	0.811	1.936(1.453–2.546)^***^	1.263(1.189–1.423)^*^	0.002	1.498(0.823–1.893)	1.318(0.697–1.894)	0.885	0.532(0.327–0.773)^*^	0.728(0.626–1.325)	0.022

AOR (95% CI) was adjusted for age, pre-pregnancy BMI, gestational weight gain, parity, IVF, caesarean history and abortion history by multivariate analyses

*GDM* Gestational Diabetes Mellitus, *TC* Total cholesterol, *TG* Triglyceride, *LDL* Low density lipoprotein, *HDL* High density lipoprotein, *AOR* Adjusted odds ratio, *PROM* Premature rupture of the membranes, *PPH* Postpartum hemorrhage, *LGA* Large for gestational age, *SGA* Small for gestational age, *LBW* Low birth weight, *NUD* Neonatal unit admission. *** *p* < 0.001, ** *p* < 0.01, *p < 0.05,

*P* value for the interaction to compare the relative risk between GDM and non-GDM women

### Association between maternal lipid profiles in the third trimester and adverse perinatal outcomes among women with GDM and non-GDM

We also evaluated the effects of the third-trimester lipid profiles of women with GDM and non-GDM on adverse perinatal outcomes (Table 4). Significant positive associations were observed between increased third-trimester TG of women with GDM and the risk of cesarean delivery (AOR = 1.834, 95% CI: 1.453–2.019, *p* < 0.001), LGA (AOR = 2.326, 95% CI: 1.728–2.914, *p* < 0.001), macrosomia (AOR = 2.032, 95% CI: 1.503–2.627, *p* < 0.001), and NUD (AOR = 1.993, 95% CI: 1.724–2.517, *p* < 0.001), which were higher than the relative risks of these perinatal outcomes in women with non-GDM. Moreover, every unit increase of HDL concentrations in the third trimester was associated with a decreased risk of cesarean delivery (AOR = 0.527, 95% CI: 0.413–0.783, *p* = 0,014), LGA (AOR = 0.525, 95% CI: 0.319–0.832, *p* = 0.017), and NUD (AOR = 0.319, 95% CI: 0.193–0.508, *p* < 0.001) in women with GDM, but only associated with a reduced risk of NUD (AOR = 0.519, 95% CI: 0.264–0.728, *p* = 0.015) in women with non-GDM. Consistent with the second-trimester analysis results, the third-trimester TC and LDL levels of women with GDM and non-GDM were not significantly associated with the included perinatal outcomes.

**Table 4:** Association of maternal lipid profile in third trimester of pregnancy with adverse pregnancy outcomes among GDM and non-GDM group

	TC, AOR (95%CI)			TG, AOR (95%CI)			LDL, AOR (95%CI)			HDL, AOR (95%CI)		
	GDM-group	non-GDM-group	*P*	GDM-group	non-GDM-group	*P*	GDM-group	non-GDM-group	*P*	GDM-group	non-GDM-group	*P*
Maternal outcome												
Caesarean delivery	1.212(0.848–1.525)	0.923(0.741–1.532)	0.773	1.834(1.453–2.019)^***^	1.229(1.087–2.314)^*^	0.001	1.137(0.748–1.327)	0.928(0.729–1.518)	0.000	0.527(0.413–0.783)^*^	0.823(0.612–1.276)	0.011
PROM	1.324(0.759–1.594)	1.014(0.715–1.578)	0.687	1.313(0.819–1.713)	0.965(0.745–1.918)	0.473	0.683(0.457–1.248)	1.182(0.689–1.563)	0.293	0.823(0.419–1.364)	1.319(0.612–1.186)	0.209
Preterm birth	1.213(0.693–1.712)	0.859(0.699–1.325)	0.628	1.322(0.812–1.921)	1.019(0.819–1.873)	0.801	0.789(0.479–1.469)	1.218(0.824–1.921)	0.512	0.722(0.335–1.846)	0.935(0.427–2.161)	0.785
Abruptio placentae	1.213(0.646–2.535)	0.911(0.657–1.984)	0.594	0.689(0.387–1.312)	1.124(0.549–1.385)	0.348	0.826(0.319–2.294)	0.994(0.512–1.946)	0.902	0.387(0.124–1.517)	0.715(0.252–1.197)	0.401
PPH	0.732(0.486–1.197)	1.235(0.764–1.597)	0.546	1.209(0.894–1.425)	1.321(0.873–1.529)	0.879	1.237(0.739–2.152)	1.253(0.728–2.093)	0.989	1.542(0.579–3.294)	1.127(0.534–2.754)	0.695
Neonatal outcome												
LGA	1.026(0.785–1.461)	0.919(0.748–1.643)	0.801	2.326(1.728–2.914)^***^	1.393(1.102–2.003)^**^	< 0.001	1.303(0.736–1.748)	0.969(0.515–1.594)	0.321	0.525(0.319–0.832)^*^	0.659(0.423–1.269)	0.015
SGA	0.797(0.623–1.713)	1.129(0.839–1.732)	0.782	1.039(0.826–1.549)	0.783(0.594–1.798)	0.733	1.785(0.549–5.593)	1.547(0.769–3.652)	0.794	0.993(0.232–3.894)	1.065(0.216–3.268)	0.879
Macrosomia	0.848(0.579–1.521)	0.872(0.528–1.397)	0.966	2.032(1.503–2.627)^***^	1.179(1.012–1.977)^*^	< 0.001	0.793(0.449–1.583)	1.081(0.546–1.626)	0.734	1.023(0.543–1.973)	1.203(0.326–1.959)	0.801
LBW	0.632(0.236–1.818)	0.836(0.364–2.014)	0.764	0.945(0.725–1.703)	0.795(0.537–1.892)	0.738	2.136(0.648–6.259)	1.796(0.386–4.287)	0.415	0.498(0.138–3.567)	0.847(0.458–1.896)	0.193
NUD	1.469(0.597–1.799)	1.218(0.516–1.862)	0.761	1.993(1.724–2.517)^***^	1.221(1.098–1.983)^*^	< 0.001	1.409(0.795–1.849)	0.819(0.516–1.682)	0.356	0.319(0.193–0.508)^***^	0.519(0.264–0.728)^*^	0.006

AOR (95% CI) was adjusted for age, pre-pregnancy BMI, gestational weight gain, parity, IVF, caesarean history and abortion history by multivariate analyses

*GDM* Gestational Diabetes Mellitus, *TC* Total cholesterol, *TG* Triglyceride, *LDL* Low density lipoprotein, *HDL* High density lipoprotein; *AOR* Adjusted odds ratio, PROM Premature rupture of the membranes, *PPH* Postpartum hemorrhage, *LGA* Large for gestational age, *SGA* Small for gestational age, *LBW* Low birth weight, *NUD* Neonatal unit admission. *** *p* < 0.001, ** *p* < 0.01, *p < 0.05,

*P* value for the interaction to compare the relative risk of outcome between GDM and non-GDM women

## Discussion

GDM, a common pregnancy complication, has been reported to be related to maternal dyslipidemia [33, 34]. Although previous studies have compared maternal lipid profiles in GDM and normal pregnancies, the results have been contradictory [25]. In this retrospective cohort study, we analyzed alterations in blood lipid profiles in the second and third trimesters of pregnancy with GDM and non-GDM. Our results showed maternal serum TC, LDL, TG, and HDL levels increased as the pregnancy advanced. Furthermore, in this study, the women with GDM had significantly higher TC and TG levels in the second and third trimesters than non-GDM. In contrast, HDL levels decreased in women with GDM, consistent with previous reports [21, 23]. However, previous studies have indicated no significant difference in serum TC, HDL, and LDL levels between women with GDM and non-GDM [25, 26, 35]. Lipid metabolism during pregnancy may be influenced by many factors, including pre-pregnancy BMI, age, diet, region, and race, which may cause differences [36].

We then comprehensively investigated the association between maternal changes in the lipid profiles and adverse perinatal outcomes in the second and third trimesters of participants with GDM and non-GDM. Recent studies have reported the correlation between maternal changes in lipid profiles in the second and third trimesters of pregnancy with adverse perinatal outcomes, including reports that second and third-trimester maternal lipid profiles in normal pregnancies were significantly correlated with LGA and macrosomia [37–39]. Moreover, it has been reported that maternal lipid profiles were related to the risk of macrosomia in non-GDM pregnancies [27]. Previous studies also showed that TG levels in GDM pregnancies positively relate to neonatal birth weight, especially as an independent predictor for LGA [40]. However, there is limited research on dyslipidemia's influence on adverse perinatal outcomes in the second and third trimesters of GDM and non-GDM pregnancies. We found that TG levels in both the second and third trimesters of pregnancy with GDM increased the risk of cesarean section, LGA, macrosomia, and NUD, the risks of which were also higher than the relative risks of these perinatal outcomes in women with non-GDM.

Furthermore, our results showed that increased HDL levels in the second and third trimesters had an inverse relationship with LGA and NUD in women with GDM, while they only had an inverse relationship with LGA in women with non-GDM. Moreover, third-trimester HDL was connected with a decreased risk of cesarean delivery in GDM pregnancies. A negative correlation between HDL concentration and neonatal birth weight among women with normal weight or those with overweight and obesity has been reported. The third trimester HDL in normal pregnancy was reported to be a stable predictor of LGA, although limited studies exist on pregnancies with GDM [37, 41]. Slagjana et al. reported that decreased HDL levels in pregnancies with GDM were related to LGA, consistent with our results. Nevertheless, the mechanism underlying the influence of HDL levels on neonatal birth weight is unclear. Additionally, TC was associated with larger neonatal sizes, and increased levels of TC and LDL at 15–27 weeks of pregnancy were related to a higher risk of preterm delivery [42]. However, in GDM and non-GDM pregnancies, we did not find an association between TC or LDL concentrations in the second and third trimesters and the adverse perinatal outcomes studied. Our findings, particularly regarding HDL and TG levels, may suggest that clinicians should closely monitor blood lipid levels of both second and third trimesters in pregnancies, especially in GDM cases.

This study had several limitations. First, the study was retrospective in design, and unmeasured confounders, including pre-gestational lipid levels, physical activity, diet, smoking, and other factors during pregnancy, were not studied. Second, our study collected serum time points that were limited to a certain gestational age in the second (24–28 gestational weeks) and third trimesters (32–36 gestational weeks), which may not reflect the serum lipid profiles throughout pregnancy or the correlation between blood lipid profiles and perinatal outcomes. Third, certain maternal clinical features (such as GWG and pre-pregnancy weight) were self-reported and may have been subject to recall bias. Multicenter prospective studies are warranted to elucidate further the correlation between maternal changes in the lipid profiles, maternal lifestyle habits, and perinatal outcomes.

## Conclusions

Our study demonstrated the correlation between maternal changes in the lipid profiles in the second and third trimesters of pregnancies with GDM and non-GDM and adverse perinatal outcomes for mothers and newborns. It further emphasized the importance of closely monitoring the blood lipids level of these pregnancies, especially in GDM, to reduce adverse perinatal outcomes and improve clinical outcomes. Moreover, prospective and multicenter clinical investigations are necessary to deeply elucidate the underlying association between maternal changes in the lipid profiles in women with GDM and adverse perinatal outcomes.

## Supplementary Information

**Additional file 1: Supplementary Table 1.** PpBMI classification, and IOM guidelines for GWG during pregnancy

## Data Availability

The data supporting this study's findings are available on request from the corresponding author. The data are not publicly available due to privacy or ethical restrictions.

## Abbreviations

GDM: Gestational diabetes mellitus
TC: Total cholesterol
TG: Triglycerides
LDL: Low-density lipoprotein
HDL: High-density lipoprotein
AOR: Adjusted odds ratio
LGA: Large for gestational age
SGA: Small for gestational age
NUD: Neonatal unit admission
GWG: Gestational weight gain
HBV: Hepatitis B virus
HCV: Hepatitis C virus
HIV: Human immunodeficiency virus
ppBMI: Pre pregnancy body mass index
IVF: In vitro fertilization
OGTT: Oral glucose tolerance test
PROM: Premature rupture of membrane
SD: Standard deviation
IQR: Interquartile range
