# Supplementary material for: Association between second- and third-trimester maternal lipid profiles and adverse perinatal outcomes among women with GDM and non-GDM: a retrospective cohort study
Source: BMC Pregnancy Childbirth. 2023 May 5;23:318. doi: 10.1186/s12884-023-05630-5 (PMC10161404; doi:10.1186/s12884-023-05630-5)
Supplement: Supplementary file 1 — Additional file 1: Supplementary Table 1. PpBMI classification, and IOM guidelines for GWG during pregnancy [file 12884_2023_5630_MOESM1_ESM.docx]

Supplementary Table 1 PpBMI classification, and IOM guidelines for GWG during pregnancy

| ppBMI classification | GWG categories | | | |
| --- | --- | --- | --- | --- |
|  | Appropriate (kg) | Inappropriate (kg) | | Excessive (kg) |
| Underweight (< 18.5 kg/m^2^) | <12.5 | 12.5-18.0 | >18.0 | |
| Normal weight (18.5-24.9 kg/m^2^) | <11.5 | 11.5-16.0 | >16.0 | |
| Overweight (25.0-29.9 kg/m^2^) | < 7.0 | 7.0-11.5. | >11.5 | |
| Obese (≥ 30.0 kg/m^2^) | < 5.0 | 5.0-9.0 | >9.0 | |

IOM, Institute of Medicine; GWG, gestational weight gain; ppBMI, pre-pregnancy Body

Mass Index
